# PACS-Integrated Tools for Peritumoral Edema Volumetrics Provide Additional Information to RANO-BM-Based Assessment of Lung Cancer Brain Metastases after Stereotactic Radiotherapy: A Pilot Study

**DOI:** 10.3390/cancers15194822

**Published:** 2023-09-30

**Authors:** Manpreet Kaur, Gabriel Cassinelli Petersen, Leon Jekel, Marc von Reppert, Sunitha Varghese, Irene Dixe de Oliveira Santo, Arman Avesta, Sanjay Aneja, Antonio Omuro, Veronica Chiang, Mariam Aboian

**Affiliations:** 1 Department of Radiology and Biomedical Imaging, Yale School of Medicine, New Haven, CT 06510, USA; nancy_ka@outlook.de (M.K.); leon.jekel@yale.edu (L.J.);; 2 Medical Faculty, Ludwig-Maximilians-University of Munich, 80336 Munich, Germany; 3 Department of Neurosurgery, Yale University School of Medicine, New Haven, CT 06510, USA; 4 Department of Therapeutic Radiology, Yale University School of Medicine, New Haven, CT 06510, USAsanjay.aneja@yale.edu (S.A.); 5 Department of Neurology, Yale University School of Medicine, New Haven, CT 06510, USA

**Keywords:** brain metastases, radiosurgery, peritumoral edema, volumetric changes to stereotactic radiotherapy, volumetric segmentation

## Abstract

**Simple Summary:**

Peritumoral edema can contribute significantly to the development of neurological symptoms in patients with brain metastases (METS), but the quantification of edema has historically been challenging. PACS-based peritumoral edema volume measurement is feasible, and this study suggests that tracking edema volume may facilitate better prediction of treatment outcome. This need is highlighted in our study as over half of the METS studied do not show congruent changes when comparing peritumoral edema volume changes to changes in diameter measurements of contrast-enhancing lesions in longitudinal assessment. Additionally, our results indicate that changes in peritumoral edema volume can pre-date tumor core size changes and could help with early identification of lesions progressing after treatment. Availability of PACS-integrated segmentation tools will allow the incorporation of edema and tumor core volumetrics into treatment response assessment in clinical practice.

**Abstract:**

Stereotactic radiotherapy (SRT) is the standard of care treatment for brain metastases (METS) today. Nevertheless, there is limited understanding of how posttreatment lesional volumetric changes may assist prediction of lesional outcome. This is partly due to the paucity of volumetric segmentation tools. Edema alone can cause significant clinical symptoms and, therefore, needs independent study along with standard measurements of contrast-enhancing tumors. In this study, we aimed to compare volumetric changes of edema to RANO-BM-based measurements of contrast-enhancing lesion size. Patients with NSCLC METS ≥10 mm on post-contrast T1-weighted image and treated with SRT had measurements for up to seven follow-up scans using a PACS-integrated tool segmenting the peritumoral FLAIR hyperintense volume. Two-dimensional contrast-enhancing and volumetric edema changes were compared by creating treatment response curves. Fifty NSCLC METS were included in the study. The initial median peritumoral edema volume post-SRT relative to pre-SRT baseline was 37% (IQR 8–114%). Most of the lesions with edema volume reduction post-SRT experienced no increase in edema during the study. In over 50% of METS, the pattern of edema volume change was different than the pattern of contrast-enhancing lesion change at different timepoints, which was defined as incongruent. Lesions demonstrating incongruence at the first follow-up were more likely to progress subsequently. Therefore, edema assessment of METS post-SRT provides critical additional information to RANO-BM.

## 1. Introduction

Brain metastases (METS) account for most of the intracranial malignancies in adults and 39%–56% originate from lung cancer [1,2] with non-small cell lung cancer (NSCLC) being the leading histological subtype [3]. Stereotactic radiotherapy (SRT), rather than whole brain radiation therapy, is now the standard of care to locally treat METS [4]. However, treatment with SRT alone is associated with a higher risk of local and/or distant tumor recurrence, making follow-up MRI surveillance crucial in these cases [5,6]. While serial imaging allows for the detection of the development of new metastases, it also offers an opportunity to study changes in SRT-treated lesions, which may allow for better prediction of local lesional outcome. For radiographical response assessment of METS, the current reference standard is outlined by the RANO-BM criteria: [7] lesions are measured in two dimensions and a minimum axial diameter of 10 mm and a perpendicular ≥5 mm are necessary to define a lesion as “measurable” [7]. The longest axial diameters (LD) in up to five lesions are added up as a summary measure, and the longitudinal change in the sum of LD, along with the patient’s clinical status and use of corticosteroids, are finally used to define neuroradiological treatment response [7]. However, in similarity to other diameter-based neuroimaging response criteria, this method shows several limitations: while summation of LD offers a summary measure, it neglects the fact that individual lesions in a single patient may behave heterogeneously after SRT and, therefore, may need to be treated differentially [8]. Additionally, 2D measurements may have significant inter-reader variability since different radiologists may use different slices to measure the same lesion [9]. Finally, two-dimensional measurements on a single imaging slice fail to depict the complex three-dimensional geometry of a tumor, which may result in insufficient grading of treatment response [9].

Volumetric segmentations of the entire tumor using every available imaging slice are potentially less prone to measurement variability [9,10], resulting in more accurate and reproducible measurements. However, volumetric treatment response assessment in clinical practice has been limited due to a lack of workflow-integrated tools for volumetric measurements, making them laborious and difficult to implement into routine neuroradiology and -oncology clinical practice. As a result, there has been limited understanding regarding the posttreatment volumetric changes of METS. Even more lacking is any understanding of changes in peritumoral edema around METS. Aside from direct tumor control, the resolution of peritumoral edema is often associated with an improvement in neurological function and possibly a better quality of life [11] and could be an equally important factor in addition to the assessment of local tumor control. This lack of data impedes the definition of volumetric response and progression criteria, which could have great potential for clinical practice and are often specifically requested by neuro-oncology providers.

In this study, we aimed to better understand the volumetric changes of perilesional edema over time after SRT and its correlation to contrast-enhancing lesion changes. For the contrast-enhancing lesion size assessment, two-dimensional-based measurements were used to facilitate the comparison of edema volume changes to the standard guidelines as per RANO-BM. To better understand the relationship between post-SRT peritumoral edema and contrast-enhancing lesion changes, a tool integrated in the picture archiving and communication system (PACS) was leveraged to longitudinally track edema volume and contrast-enhancing lesion size.

## 2. Methods

### 2.1. Patient Selection

**Figure 1:**
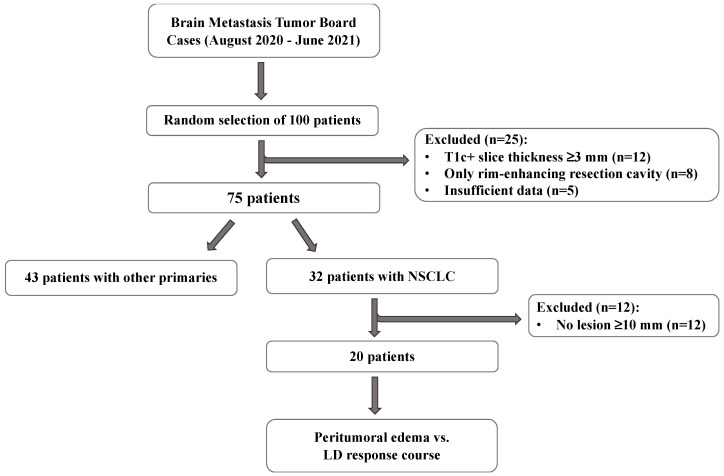
Flowchart of the patient selection process. From the initial cohort of 100 randomly selected patients, only those with non-small cell lung cancer (NSCLC) brain metastases (METS) who presented with at least one lesion with a longest diameter (LD) ≥10 mm were included in our study (T1c+: post-contrast T1-weighted).

IRB approval was obtained for this single-center study. The patient selection process is described in Figure 1. Briefly, we collected 291 cases presented at our METS tumor board meetings between August 2020 and June 2021 or identified cases using pathology report search on Montage (Nuance Communications, Inc., Burlington, MA, USA) and randomly selected 100 patients who received SRT treatment. Cases were then excluded if they (1) were missing treatment or follow-up MRIs, (2) if the METS were surgically resected, (3) if post-contrast T1-weighted (T1c+) images were obtained with slice thickness ≥3 mm and/or T2-weighted (T2w) fluid-attenuated inversion recovery (FLAIR) sequences were missing, (4) if primary cancer type was not NSCLC, and (5) if initial LD on T1c+ image on the SRT treatment scan was <10 mm.

### 2.2. Measurement of 2D Diameters of Contrast-Enhancing Lesions, and Volume of Peritumoral Edema

The 2D measurements of the contrast-enhancing lesion and the volumetric segmentations of peritumoral edema were performed using PACS-integrated tools on Visage7 (Visage Imaging Inc., San Diego, CA, USA) (Figure S1). All trainee measurements performed were revised by a board-certified neuroradiologist.

For 2D measurements of contrast-enhancing lesions, all intraparenchymal METS were measured on the first SRT MRI scan on T1c+ sequences. The workflow of 2D lesion tracking using Visage7 has been described by Cassinelli Petersen G. et al. [8]. The peritumoral volume of contrast-enhancing lesions with LD ≥10 mm was segmented by delineating the FLAIR hyperintense region surrounding and including the tumor core and edema using a PACS-integrated “Balloon tool” (Figure S1). The volumetric segmentations were performed manually on every single imaging slice. Initial peritumoral edema segmentations were performed on T2w-FLAIR 0–30 days prior to SRT treatment, since the T2w-FLAIR sequence is missing on intraprocedural scans. All patients were tracked for up to seven follow-up scans at intervals of 6–12 weeks.

### 2.3. Data Analysis

GraphPad Prism version 9.2 for macOS (GraphPad Software, San Diego, CA, USA) was used to analyze the data. MRIs at 0–90, 91–180, 181–270, 270–365, and >365 days after SRT treatment were considered. Measurements on the day of SRT (2D measurements for tumor core) or 0–30 days prior (peritumoral volumetric measurements) were defined as baseline. Tumors with coalescent peritumoral edema were excluded from the analysis as edema cannot be assigned to one specific tumor core on T1c+. To compare posttreatment courses of LD of contrast-enhancing lesions and volume of peritumoral edema, response curves were created. Wilcoxon signed-rank test was used to compare pre- and posttreatment changes in peritumoral edema and contrast-enhancing lesion size (α = 0.05). To evaluate the difference between the relative peritumoral edema volume change between lesions demonstrating an increase in edema and lesions demonstrating a decrease in edema during assessment, a two-tailed Mann-Whitney U test (α = 0.05) was used. The total number of lesions, lesions per patient, lesions with LD ≥10 mm per patient, and mean time of follow-up were calculated. A two-tailed Mann-Whitney U test (α = 0.05) was performed to compare these values between the group of patients that remained alive and the group that deceased during the time of assessment.

### 2.4. Comparing LD of Contrast-Enhancing Lesions with Volume of Peritumoral Edema

Contrast-enhancing lesions and peritumoral edema response curves were qualitatively compared at each post-SRT time point. Response curves were defined as congruent if the diameter of tumor core and the volume of peritumoral edema were concurrently increasing, decreasing, or completely remitted. Otherwise, they were labeled as incongruent. If new lesions appeared during the time of assessment, prior reports were checked for the presence of a FLAIR hyperintense signal as an early predictor of lesion appearance. Spearman’s rank correlation was used to evaluate the relationship between the course of LD of contrast-enhancing lesion and the volume of peritumoral edema (α = 0.05).

To classify METS that showed congruent or incongruent response curves, each lesion was further categorized analogously to RANO-BM criteria [7]. Partial response was defined as a decrease ≥30% in LD from baseline, progressive disease as an increase ≥20% in LD from nadir, and complete response when the lesion disappeared in the follow-up scan. When a lesion occurred that was not present on a prior MRI, it was classified as a new lesion, and when no criterion applied, lesions were defined as stable disease [7].

We further looked at the initial SRT response of METS at the first follow-up, classifying the tumor core into the different response categories according to RANO-BM and qualitatively defining the peritumoral edema as increasing, decreasing, or completely remitted.

### 2.5. Steroid Admission and Peritumoral Edema Course

We additionally obtained clinical information about steroid use during the time of SRT intervention for descriptive characterization.

## 3. Results

### 3.1. Patient Characteristics

From the initial cohort of 100 randomly selected patients, 20 patients with NSCLC METS who presented with at least one lesion with an LD ≥10 mm were included in our study. A detailed account of the characteristics of the patients included in our study can be found in Scheme S1. Briefly, the selected 20 patients (mean age ± standard deviation (SD) = 59.4 ± 10.3 years, female-to-male ratio = 11:9) presented with a total of 169 METS, and a median of four lesions per patient (interquartile range (IQR) 3–10). Of the included patients, 90% had multicentric and 10% had monocentric METS. The median number of lesions with an LD ≥10 mm was two (IQR 1–3, n of lesions = 50), with nine of those lesions showing a confluent peritumoral edema. Half of the patients died prior to their seventh follow-up MR scan. The mean ± SD follow-up time post-SRT for those patients was 221.5 ± 80.2 days, and for patients who were alive during the whole follow-up assessment, it was 479.9 ± 144.6 days (*p* = 0.0001). The median number of scans available for the study was five (IQR 4–6) in the alive cohort and 3.5 (IQR 3–5) in the dead cohort. There was no significant difference in total number of lesions (*p* = 0.69) or number of lesions with an LD ≥10 mm (*p* = 0.93) between the group of patients that remained alive and the group that died during follow-up.

### 3.2. LD Assessment of Contrast-Enhancing Tumor Core

The median pretreatment LD of contrast-enhancing tumor core measured 14 mm (IQR 11–17 mm, n of lesions = 35).

A significant decrease was measured between the median pre- and posttreatment LD of contrast-enhancing tumor core at 0–90 days (*p* < 0.0001) and between 91–180 and 181–270 days (*p* = 0.02) post-SRT. No significant difference could be measured between the other time points post-SRT (Scheme S2). No significant difference could be identified regarding the median LD of contrast-enhancing tumor core between the group of patients that deceased and the group that remained alive for the pretreatment and for any of the follow-up assessment time periods. The median LD of contrast-enhancing tumor core over follow-up time is depicted in Figure S2.

Further, we calculated the median relative LD change of the contrast-enhancing lesions relative to the pre-SRT baseline. The median relative LD change post-SRT was 71% at 0–90 days (IQR 46–81%, n of lesions = 31), 43% at 91–180 days (IQR 26–67%, n of lesions = 30), 45% at 181–270 days (IQR 6.3–78.8%, n of lesions = 16), 35.5% at 271–365 days (IQR 5–50% n of lesions = 16), and 25% at >365 days (IQR 0–66.7%, n of lesions = 14). There was no significant difference between the median relative LD change of contrast-enhancing tumor core at initial follow-up (0–90 days) for lesions progressing and lesions decreasing in LD size (*p* = 0.12).

### 3.3. Volumetric Assessment of Peritumoral Edema

**Scheme 1:**
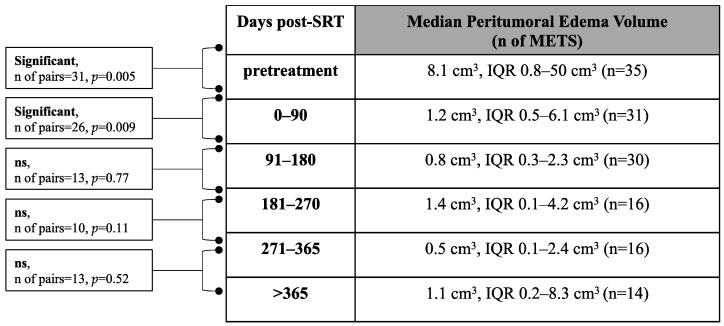
Median peritumoral edema volume over follow-up time. Median peritumoral edema volume at pretreatment and at different post-SRT time points. (Wilcoxon signed-rank test, *p* < 0.05) (SRT: Stereotactic radiotherapy, n: number, ns: not significant, IQR: interquartile range).

The median pretreatment peritumoral edema volume was 8.1 cm^3^ (IQR 0.8–50 cm^3^, n of lesions = 35) (Scheme 1). A significant decrease was measured between the pre- and posttreatment peritumoral edema volume at 0–90 days (*p* = 0.005) and between 0–90 and 91–180 days (*p* = 0.009) post-SRT. No significant difference could be measured between the other follow-up time points (Scheme 1). No significant (*p* = 0.66) difference could be measured regarding the pretreatment median peritumoral edema volume for the group of patients that died (median 8.1 cm^3^, IQR 0.8–32.6 cm^3^, n of lesions = 19) and the group that remained alive (median 9 cm^3^, IQR 0.8–64.2 cm^3^, n of lesions = 16).

We also looked at the median relative peritumoral edema volume change during assessment. The median relative peritumoral edema volume post-SRT was 37% at 0–90 days (IQR 8–114%, n of lesions = 31), 11.5% at 91–180 days (IQR 2–55.8%, n of lesions = 30), 24.5% at 181–270 days (IQR 1–94.5%, n of lesions = 16), 2.5% at 271–365 days (IQR 1.3–18.3% n of lesions = 16), and 6.5% at >365 days (IQR 1–38%, n of lesions = 14).

Median relative peritumoral edema volume change at the initial follow-up (0–90 days) was significantly different between lesions progressing and lesions decreasing in edema volume during the time of the study (*p* < 0.0001). At the first follow-up, lesions increasing had a median volume increase of 14% and lesions shrinking in edema had a median volume reduction of 89%.

**Figure 2:**
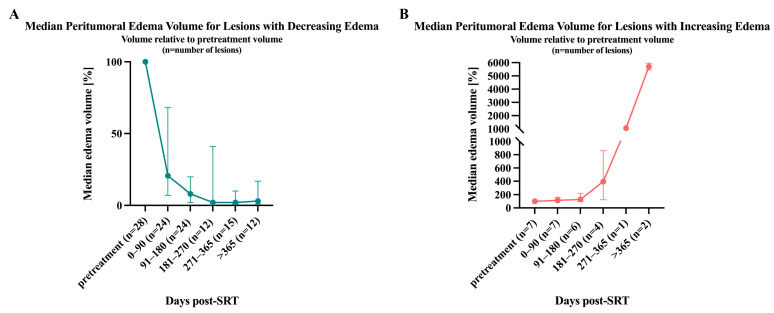
Median relative peritumoral edema volume change over follow-up time. Median peritumoral edema volume change and the IQR are depicted relative to the pretreatment volume. (**A**) Median relative edema volume for lesions decreasing during the time of the study. (**B**) Median relative edema volume for lesions progressing over follow-up time.

Lesions with increasing peritumoral edema (n = 7/31) showed a nearly continuous growth in the median relative edema volume post-SRT (114% at 0–90 days, 129.5% at 91–180 days, 395.5% at 181–270 days, 1059% at 271–365 days and 5705% at >365 days) (Figure 2B). Interestingly, all lesions that experienced progress in edema for the duration of this study showed an increase in edema volume at the first follow-up time point.

For lesions with decreasing peritumoral edema volume (n = 24), a similar and almost continuous reduction in median relative edema size could be determined (20.5% at 0–90 days, 8% at 91–180 days, 2% at 181–270 days, 2% at 271–365 days, and 3% at >365 days) (Figure 2A). During this study, 3/24 lesions experienced a complete remission of peritumoral edema.

Only 4/24 lesions that showed a decreasing peritumoral edema volume showed a paradoxical increase at the first post-SRT scan but decreased at the second follow-up. Some lesions (n = 6/24) that declined in peritumoral edema did not show a continuous decrease, but rather a more waxing and waning type of course: Half of those lesions (n = 3/6) experienced a regrowth in peritumoral edema volume at the second follow-up, and the other half at the third follow-up. The volume of peritumoral edema volume for those lesions was never greater than the initial pretreatment volume.

### 3.4. Correlation of Tumor Core LD Versus Peritumoral Edema Volume

**Figure 3:**
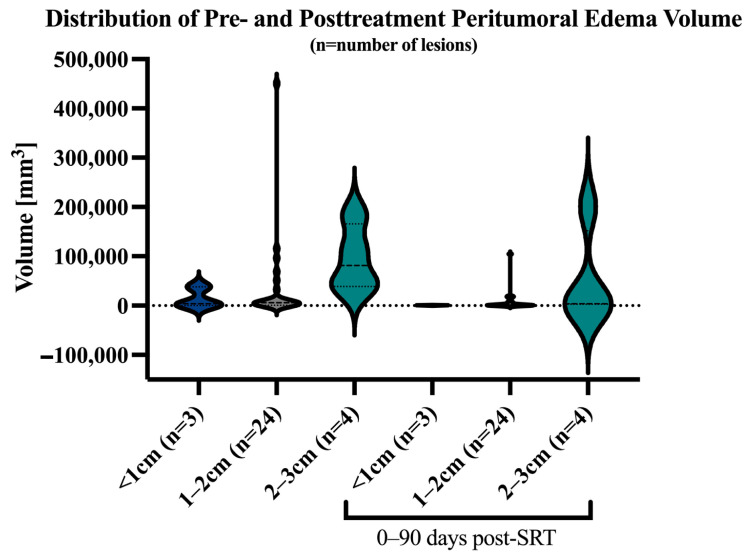
Violin plot with distribution of peritumoral edema stratified by baseline LD of contrast-enhancing tumor core. Pretreatment median peritumoral volume for lesions with LD <1 cm was 3.9 cm^3^ (IQR 0.4–38.1 cm^3^), 5.9 cm^3^ (IQR 1–27.4 cm^3^) for LD 1–2 cm, and 81 cm^3^ (IQR 38.9–166 cm^3^) for LD 2–3 cm. The median peritumoral edema volume decreased post-SRT to 0.5 cm^3^ (IQR 0.3–0.9 cm^3^) for LD <1 cm, 1.1 cm^3^ (IQR 0.5–7.1 cm^3^) for LD 1–2 cm, and 3.8 cm^3^ (IQR 2.7–152 cm^3^) for LD 2–3 cm.

A significant moderate positive correlation could be computed between the LD of tumor core and the peritumoral edema volume (r_s_ = 0.75, *p* < 0.0001, n of pairs = 169), although as can be seen in Figure 3, there can be significant variability across the ratio of LD to peritumoral edema volume both at baseline and within 90 days post-SRT.

**Figure 4:**
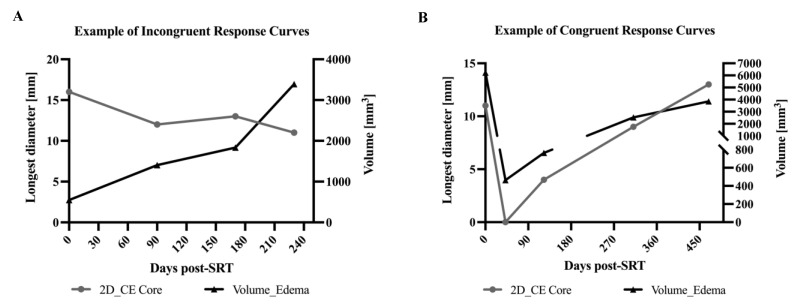
Example of treatment response curves for contrast-enhancing tumor core and peritumoral edema volume. (**A**) Exemplary case with incongruent response curves. The volume for peritumoral edema increased over the follow-up period, whereas the LD of contrast-enhancing tumor core showed a waxing and waning type of course with a decrease at the last follow-up at 230 days after SRT. (**B**) Exemplary case with congruent response curves. Both peritumoral edema volume and LD of contrast-enhancing tumor core first decreased in size post-SRT. From 45 days post-SRT, both response curves increased during the whole period of follow-up. (CE: contrast-enhancing).

**Figure 5:**
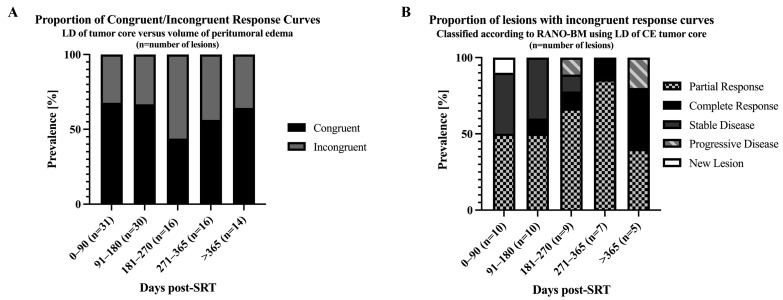
Proportion of congruent/incongruent treatment response curves for LD of contrast-enhancing tumor core and peritumoral edema volume. METS were categorized as congruent if the course of response curves for LD of contrast-enhancing tumor core and volume of peritumoral edema were both increasing, decreasing, or completely remitted; otherwise, they were defined as incongruent. (**A**) Congruence to incongruence ratio for peritumoral edema volume/LD for contrast-enhancing tumor core ranged from 68/32 to 44/56 over follow-up time. (**B**) Lesions with incongruent response curves were further classified according to RANO-BM criteria using the LD of contrast-enhancing tumor core.

We additionally analyzed treatment response curves based on binary evaluation of whether the curves for lesion core size to peritumoral edema volume are congruent or incongruent (Figure 4). Despite the computed positive correlation, the LD and the peritumoral edema changes post-SRT did not always correlate. The ratio between congruent/incongruent changes for LD of tumor core and peritumoral edema volume also changed over follow-up time (Figure 5).

After SRT (0–90 days), 32% (n = 10/31) of lesions showed incongruent changes for edema volume and contrast-enhancing tumor core size. At the different follow-up time periods, the ratio for incongruently changing lesions was: 91–180 days: n = 10/30 (33%), 181–270 n = 9/16 (56%), 271–365 days n = 7/16 (44%), and >365 days n = 5/14 (35.7%) (Figure 5).

The median pretreatment edema/lesion ratio for lesions decreasing in tumor core and edema size at the last available follow-up was 905.8 (IQR 435.9–3812), and for lesions increasing in size, it was 117 (IQR 32.8–6614).

Of the 10/31 lesions (32%) demonstrating incongruent change at the first follow-up (0–90 days post-SRT), all lesions decreased in contrast-enhancing tumor core LD but increased in peritumoral edema volume: Interestingly, most of those lesions (n = 6/10) demonstrated congruent change with an increase in both the LD and the peritumoral edema volume at the last follow-up time point. Three of the incongruently changing lesions decreased in LD of tumor core and peritumoral edema volume at the last follow-up and one decreased in LD with growing peritumoral edema volume.

Lesions presenting with congruent change for LD of contrast-enhancing tumor core and peritumoral edema volume at first follow-up were mostly decreasing (n = 20/21) in edema and tumor core size: Most (85% (n = 17/20)) of those lesions showed prolonged local control at the last available follow-up, with continuous reduction in size. One lesion (n = 1/20) increased in LD of tumor core and peritumoral edema volume at the last available scan. For the remaining two lesions, no follow-up scans were available.

Only one lesion with congruent change at initial post-SRT scan (n = 1/21) experienced an increase in contrast-enhancing tumor and edema, which subsequently decreased at the last follow-up.

We further categorized the tumor core after the initial SRT as per existing RANO-BM criteria and the peritumoral edema volume change according to the qualitative assessment of the response curves. Most lesions (n = 16/31) were classified as stable disease with the majority of the lesions presenting with decreasing (n = 13/16) peritumoral edema. There was a small subset of lesions (n = 3/16) with a paradoxical increase in edema. In lesions with partial response, 60% (n = 6/10) had expected decreasing edema, while 40% (n = 4/10) had unexpected increasing edema. The remaining lesions were classified as complete response with increasing edema (n = 2/31) or decreasing edema (n = 1/31). The progressive lesion and the one new lesion had an expected increasing edema (Figure S3). No lesion showed complete resolution of edema at this point of assessment. At other time points, resolution of edema was found in lesions that demonstrated partial and complete response.

### 3.5. Effects of Steroid Treatment on Peritumoral Edema Volume

We further screened if and when patients received steroids and how the course of peritumoral edema changed according to steroid administration timing. Most patients (85%, n = 17/20) were treated with steroids at some point during assessment with most patients (65%, n = 13/20) receiving steroids shortly prior to and during the time of SRT treatment. Half of the patients (n = 9/18) were treated with steroids 0–90 days, 41% (n = 7/17) at 91–180 days, 18% (n = 2/11) at 181–270 days, and 30% (n = 3/10) at 270–365 days post-SRT treatment. No patient (n = 8) was treated with steroids >365 days after initial SRT treatment.

Shortly after SRT treatment at 0–90 days, 81% (n = 13/16) of steroid-treated METS showed a decreasing peritumoral edema volume, whereas edema increased in 19% (n = 3/16). The median relative peritumoral edema volume at 0–90 days post-SRT was 27% (IQR 6–92%, n of lesions = 19) for lesions that received steroids shortly prior to or during the procedure. For lesions that were not treated with steroids during this period, the median relative peritumoral edema volume was 113% (IQR 11–204%, n of lesions = 12).

At the second follow-up 91–180 days post-SRT, peritumoral edema volume decreased in 83% (n = 10/12) of METS and increased in 17% (n = 2/12). At 181–270 days post-SRT, one lesion demonstrated decreasing peritumoral edema, one was increasing, and one had resolution of edema. After initial SRT at 271–375 days, 67% (n = 2/3) of METS showed decreasing and 33% (n = 1/3) increasing peritumoral edema volume.

## 4. Discussion

Patients with NSCLC develop METS in up to 64% of cases [12] and often present with multiple lesions [2]. The median survival of patients with NSCLC METS is estimated between 7 and 16 months after SRT, and up to 20 months if combined with immunotherapy [13,14,15]. Survival after NSCLC METS radiosurgery is associated with different factors such as age, control of systemic disease, interval between diagnosis of lung cancer and development of METS, performance status, and neurologic deficits [13,16,17]. Clinically, patients with NSCLC METS present with neurological symptoms including seizures, headaches, and focal neurologic and cognitive impairment [18], often resulting from the peritumoral edema, as it can be severalfold larger than the contrast-enhancing tumor core [19]. While this peritumoral edema is believed to be primarily of vasogenic nature, some studies have indicated the presence of neoplastic cells extending into the edema: [20,21] One autopsy study was able to show invasive growth of 50% of solid METS reaching beyond the contrast-enhancing lesion, [20] while another study reported the reduction of local tumor recurrence after surgical resection by extending the resection cavity 5 mm from the contrast-enhancing tumor core [21]. However, as of yet, there is insufficient knowledge about the cause of peritumoral edema, its correlation to tumor volume, and its impact on survival [22,23,24,25], leading to insufficient management of perilesional edema pre-and posttreatment.

In our study, we sought to examine how the peritumoral edema behaves in relation to the contrast-enhancing tumor core after SRT by measuring the 2D diameters of tumor core and the peritumoral edema surrounding the contrast-enhancing lesion using PACS-integrated tools. Comparison between treatment response curves was performed qualitatively and by computing Spearman’s rank correlation coefficient.

First, we evaluated the changes in peritumoral edema volume after SRT. Most lesions (77%) decreased in edema size after treatment. Furthermore, our data indicates that the course of peritumoral edema volume change can be predicted within the first three months post-SRT: Interestingly, lesions experiencing regression in peritumoral edema volume at first follow-up (0–90 days) mostly continued to decrease in edema size. Solely a subset of lesions (17%) experienced a waxing and waning course: After an initial decrease in edema, a surge in edema volume was measured, followed by a final decrease. Conversely, most lesions increasing in peritumoral edema volume at the initial scan post-SRT consistently progressed during the entire follow-up period. Only 10% of lesions demonstrated a surge in perilesional edema volume at the first assessment with a subsequent decrease in edema at the second follow-up. These findings align with previous studies that reported early tumor core and edema reduction within the first three months to be associated with consequent local control [26,27,28].

Next, we compared the change of lesion core size in relation to the change in peritumoral edema volume. We were able to identify a moderate (r_s_ = 0.75) positive relationship between the LD increase of contrast-enhancing tumor core and peritumoral edema volume. A correlation between tumor enhancing size and surrounding edema has been shown previously [11,19,29], but tools to measure peritumoral edema are still not routinely available in clinical practice.

We also analyzed treatment response curves for tumor core size and peritumoral edema based on binary evaluation of whether the curves are congruent or incongruent. Strikingly, while we did find a positive correlation between edema and enhancing tumor size, we also found that up to 56% of METS showed incongruent treatment response curves at different time points of the assessment. The congruence to incongruence ratio changed over follow-up time, ranging from 68/32 to 44/56 and, therefore, no translatable rule for conversion of contrast-enhancing lesion size to peritumoral edema volume was possible.

Looking at the initial posttreatment changes, our findings indicate that lesions with increasing peritumoral edema volume were more likely to show incongruent treatment response with contrast-enhancing tumor core decreasing in size. Interestingly, examining those lesions on the last available follow-up scan, we could observe that most lesions demonstrated congruent change with both contrast-enhancing tumor core and peritumoral edema growing. In accordance with these results, lesions with decreasing peritumoral edema volume mostly presented with congruent response curves with shrinking contrast-enhancing tumor core size. Lesions demonstrating congruent change at first follow-up with a decline in size for edema and tumor core were, thus, more likely to decrease throughout the assessment. In conclusion, these findings suggest that assessing peritumoral edema and correlating changes in edema with alterations in contrast-enhancing lesion size could be beneficial to identify progressive lesions and subsequently optimize treatment regimes. It is important to consider that progression in size does not necessarily imply tumor recurrence but could also be due to treatment-related effects based on systemic or radiation therapy. Leeman et al. have demonstrated that in preoperative imaging, lesions with greater edema may be more prone to be radionecrotic [30]. Time between SRT and (re)growth may also be important. Histologically lesions resected >12 months after SRT depicted radiation-induced necrosis without tumor cells [30].

Furthermore, in our study, a pattern could be appreciated for lesions classified as progressive disease and in the case of new lesions. These were more likely to show congruent treatment response curves where tumor core and peritumoral edema both increased in size at almost all follow-ups. Response curves for new lesions mostly demonstrated congruent increase in size at the same timepoint of assessment for both the contrast-enhancing lesion and the peritumoral edema. This finding is not unexpected as, among other factors, peritumoral edema is believed to be caused by the formation of new abnormal tumor vessels that lack tight junctions [31]. As the contrast-enhancing tumor core grows, the development of new vessels is expected and triggered by the upregulation of factors like vascular endothelial growth factor (VEGF) [31]. VEGF itself also leads to an increase in vascular permeability, aggravating the formation of peritumoral edema [31]. The congruent increase in tumor size and peritumoral edema volume could, moreover, be a consequence of the decreased venous drainage [19] caused by the mass effect of the lesion and edema. Nonetheless, it needs to be kept in mind that progression of lesions after SRT could also be related to radiotherapy effects and not progression of disease itself [7]. As Patel et al. were able to confirm, about one-third of the patients with METS treated with radiosurgery showed an asymptomatic increase in tumor size, which often was related to inflammation and necrosis [32].

Assessing the initial SRT treatment response by applying the currently used RANO-BM criteria, the results also suggest that in some lesions, peritumoral edema volume does not behave congruently to the contrast-enhancing tumor core. As an example, in lesions with partial response, 60% of the lesions had expected decreasing edema, but up to 40% of lesions had paradoxically increasing edema. This could be related to systemic therapy that the patient is receiving, and further examination of the degree of edema changes is needed in future studies. We propose to grade edema in the following manner: stable edema volume (less than 25% change), decreased edema (over 25% change), increased edema (over 25% change), and resolution of edema. This new classification will help improve clinical reporting of edema volume in relation to contrast-enhancing size to provide precision assessment of treatment response. Furthermore, increasing the availability of PACS-based tools including deep learning autosegmentation algorithms [33] may allow easier implementation of this critical assessment into clinical practice.

In our study, most patients received steroids at some point during the follow-up assessment for new or worsening symptomatic edema, as they are the mainstay of treatment for this indication [12]. Patients who received steroids in our study mostly demonstrated an expected decrease in peritumoral edema volume. Peritumoral edema assessment could be helpful to determine patients who will progress in lesion size, but also in edema volume prior to corticosteroid administration. This seems important as the use of corticosteroids may compromise the effectiveness of immunotherapeutic agents such as programmed death ligand 1 (PD-(L)1) inhibitors, especially when given at the beginning of PD-(L)1 inhibitor treatment [34].

## 5. Limitations

Our study had several limitations. Given its retrospective nature, follow-up studies for some of the included patients were not available at the defined timepoints. In addition, not all studies had identical imaging protocols. Another limitation is our focus on NSCLC, the most common cause of METS [1,2], and the exclusion of metastatic brain lesions from non-NSCLC. Future work should aim to include metastases from other primary tumors to increase the generalizability of the results. Furthermore, systemic therapies, including anti-angiogenic, target, or immunotherapy, which may influence edema evolution, were not included in the analysis. The small sample size needs to be considered when interpreting the data. Lastly, studies are needed to assess the clinical relevance of the results and the influence of other variables including inflammation, radiation-induced necrosis, and/or posttreatment changes.

## 6. Conclusions

Our results may guide future efforts to improve treatment assessment by clinical and neuroradiological criteria after SRT. Given that contrast-enhancing tumor core and peritumoral edema volume do not show congruent changes in over half of the METS, the authors recommend including both the volumetric longitudinal measurements of peritumoral edema and the contrast-enhancing portion of METS in the radiographical assessment after treatment.

## Data Availability

Data are contained within the article or supplementary material.

## Supplementary Materials

The following supporting information can be downloaded at: https://www.mdpi.com/article/10.3390/cancers15194822/s1, Figure S1: PACS-Integrated Segmentations Demonstrated in an Exemplary Case; Scheme S1: Characteristics of Included Patients; Scheme S2: Median LD of Contrast-Enhancing Tumor Core; Figure S2: Median LD of Contrast-Enhancing Tumor Core Course over Follow-Up Time; Figure S3: Changes in Edema in Lesions after Initial SRT Based on Treatment Response Assessed by RANO-BM.
