# Supplementary material for: PACS-Integrated Tools for Peritumoral Edema Volumetrics Provide Additional Information to RANO-BM-Based Assessment of Lung Cancer Brain Metastases after Stereotactic Radiotherapy: A Pilot Study
Source: Cancers (Basel). 2023 Sep 30;15(19):4822. doi: 10.3390/cancers15194822 (PMC10571649; doi:10.3390/cancers15194822)
Supplement: Supplementary file 1 [file cancers-15-04822-s001.zip › cancers-2590826-supplementary.pdf]

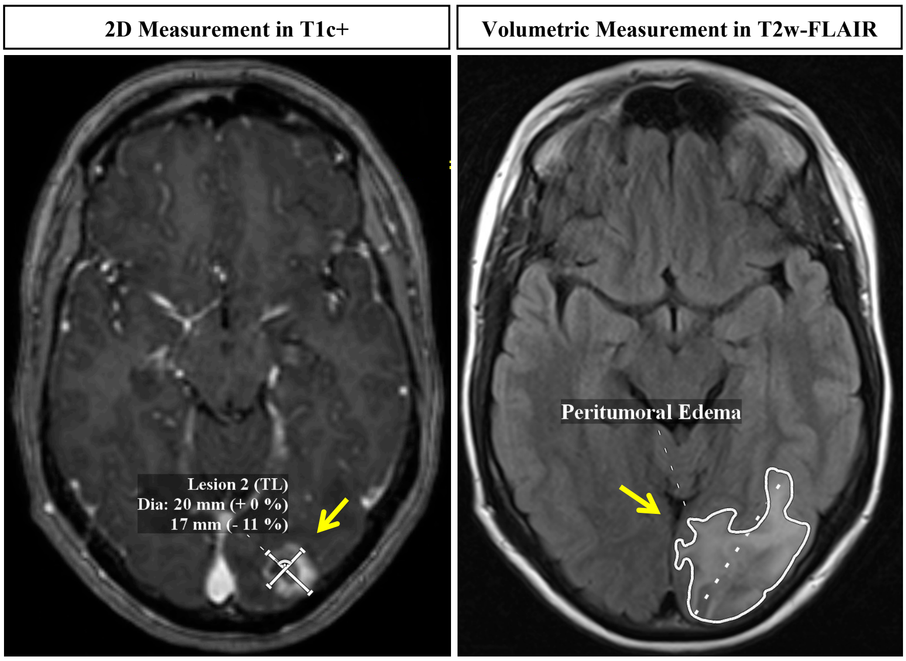

Figure S1: PACS-Integrated Segmentations Demonstrated in an Exemplary Case. MRI of the last follow-up (597 days post-SRT). The image on the left-hand side shows the 2D measurement on axial T1c+. The LD was 20 mm, and the perpendicular diameter was 17 mm. The axial T2-weighted (T2w)-fluid-attenuated inversion recovery (FLAIR) image on the right-hand side demonstrates the volumetric segmentation of the peritumoral edema. The peritumoral edema volume for this lesion was 89.6 cm^3 (TL: temporal lobe, Dia: diameter).

Scheme S1: Characteristics of Included Patients.

	All patients with NSCLC	Alive patients	Dead patients
**Total n of patients**	20	10	10
**Mean±SD age**	59.4±10.3 years	55±11.8 years	63.7±7.6 years (ns, *p*=0.06)
**F:M ratio**	11:9	5:5	6:4
**Total n of METS**	169	100	69
**Total n of METS with LD ≥10 mm**	50	26	24
**Median n of METS per patient**	4 (IQR 3-10)	4 (IQR 3-10.3)	4 (IQR 1.8-11.8) (ns, *p*=0.69)
**Median n of METS with LD ≥10 mm per patient**	2 (IQR 1-3)	2 (IQR 1-3.8)	2 (IQR 1-3.5) (ns, *p*=0.93)
**Mean±SD follow-up time post-SRT**	350.7±174.7 days	479.9±144.6 days	221.5±80.2 days (significant, *p*=0.0001)

The first column summarizes the characteristics for all patients included in our study. Columns two and three separate the information for patients that remained alive or deceased during the follow-up assessment (SD: standard deviation, F:M ratio: Female-to-Male ratio).

Scheme S2: Median LD of Contrast-Enhancing Tumor Core.

Days post-SRT	Median LD of Contrast-Enhancing Tumor Core (n of METS)
**pretreatment**	14 mm, IQR 11–17 mm (n=35)
**0–90**	9 mm, IQR 6–13 mm (n=31)
**91–180**	6.5 mm, IQR 4–13.25 mm (n=30)
**181–270**	7 mm, IQR 0.75–11.75 mm (n=16)
**271–365**	4.5 mm, IQR 0.75–9 mm (n=16)
**>365**	4 mm, IQR 0–13.25 mm (n=14)

Significant, n of pairs=31, *p*<0.0001 (pretreatment vs. 0–90)

ns, n of pairs=26, *p*=0.14 (0–90 vs. 91–180)

significant n of pairs=13, *p*=0.02 (91–180 vs. 181–270)

ns, n of pairs=10, *p*>0.99 (181–270 vs. 271–365)

ns, n of pairs=13, *p*=0.92 (271–365 vs. >365)

Median LD of **contrast-enhancing** tumor core pretreatment and at different post-SRT follow-ups. (Wilcoxon signed-rank test, p<.05).

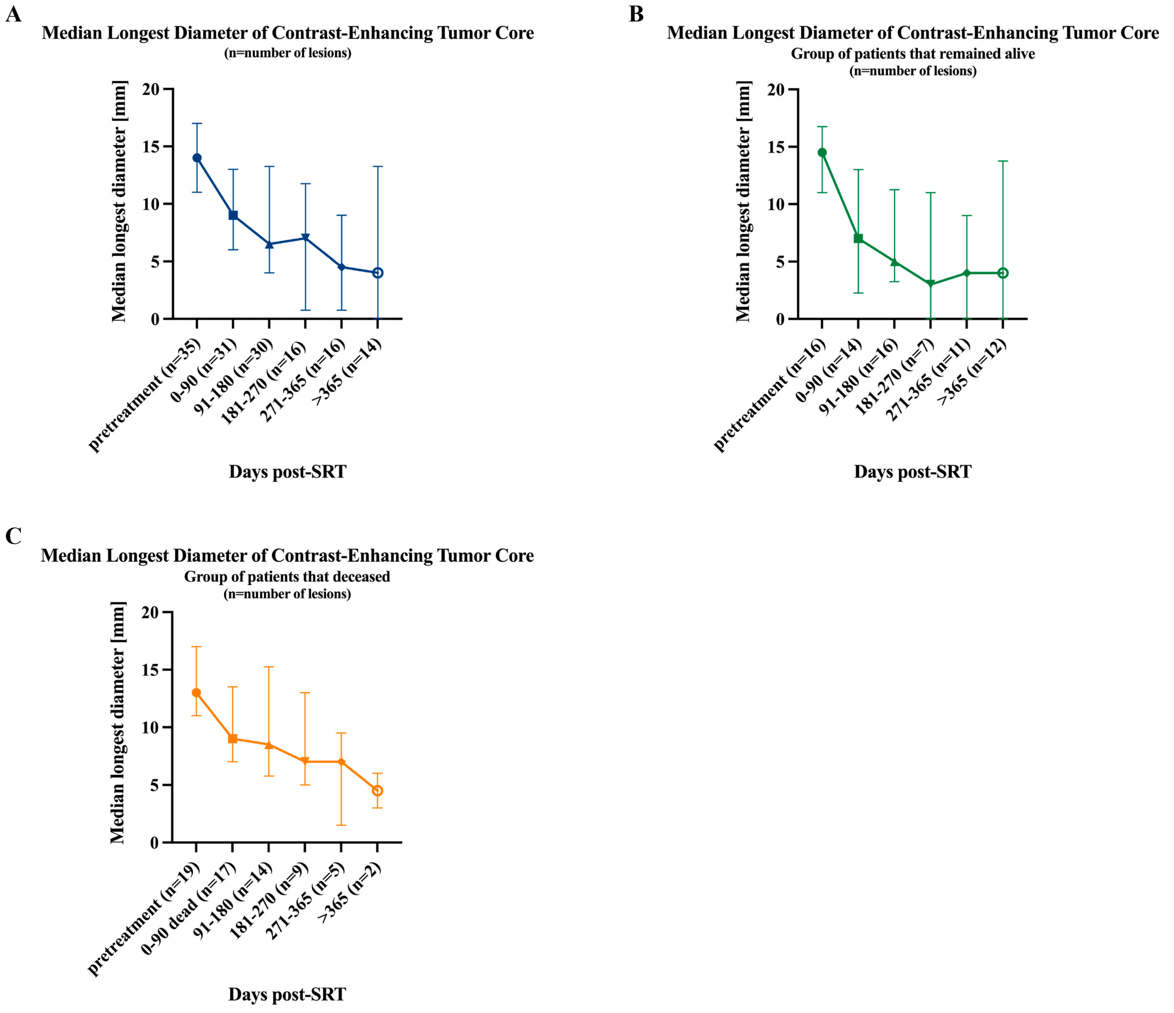

Figure S2. Median LD of Contrast-Enhancing Tumor Core Course over Follow-Up Time. The median and the IQR of the LD of **contrast-enhancing** tumor core are depicted relative to the

pretreatment LD for all included patients **(A)** and separately for the cohort of patient that remained alive **(B)** or deceased **(C)** during the period of assessment.

Inital Posttreatment Response of Tumor Core and Peritumoral Edema

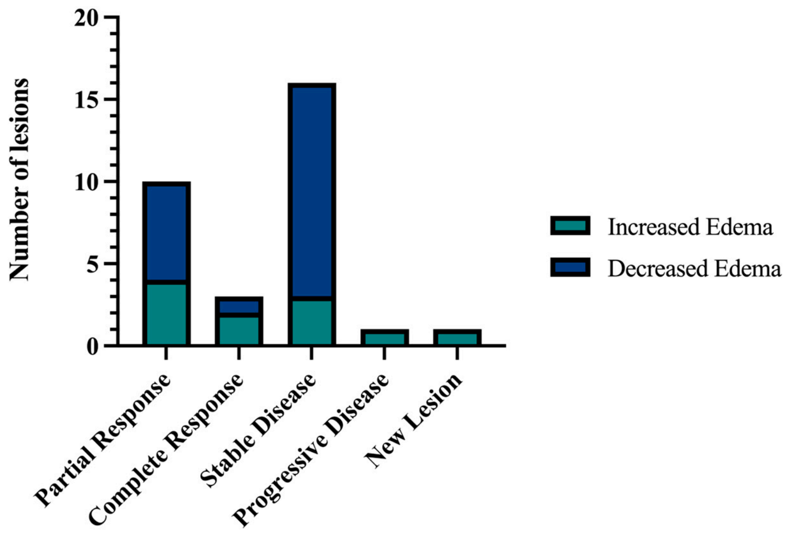

Figure S3: Changes in Edema in Lesions after Initial SRT Based on Treatment Response Assessed by RANO-BM. Lesion response assessment was performed using RANO-BM criteria on **contrast-enhancing** portion of the lesion. Edema surrounding the lesions based on response category was further assessed. In this table, lesions that demonstrate partial response based on ≥30% decrease in size of **contrast-enhancing** portion had variable edema changes, with approximately 60% of the lesions demonstrating decreased edema and 40% of the lesions demonstrating paradoxically increased edema. In lesions that demonstrated complete response, one case (33%) showed decreasing edema and 67% of cases showed paradoxically increased edema. In lesions with stable disease, majority of them had expected decrease in edema; but up to 19% showed increased edema. One lesion demonstrated progressive disease and one was classified as a new lesion with expected increase in edema. Number of lesions: n=31 for 0–90 days post-SRT.
